# Mesoporous Silica as an Alternative Vehicle to Overcome Solubility Limitations

**DOI:** 10.3390/pharmaceutics16030386

**Published:** 2024-03-12

**Authors:** Tim Becker, Jan Heitkötter, Anna K. Krome, Andrea Schiefer, Kenneth Pfarr, Alexandra Ehrens, Miriam Grosse, Birthe Sandargo, Ingo Stammberger, Marc Stadler, Marc P. Hübner, Stefan Kehraus, Achim Hoerauf, Karl G. Wagner

**Affiliations:** 1 Department of Pharmaceutical Technology and Biopharmaceutics, University of Bonn, 53121 Bonn, Germany; tim.becker@uni-bonn.de (T.B.); j.heitkoetter@uni-bonn.de (J.H.); krome@uni-bonn.de (A.K.K.); 2 German Center for Infection Research (DZIF), Partner Site Bonn-Cologne, 53127 Bonn, Germany; andrea.schiefer@uni-bonn.de (A.S.); kenneth.pfarr@ukbonn.de (K.P.); aehrens@uni-bonn.de (A.E.); huebner@uni-bonn.de (M.P.H.); skehraus@uni-bonn.de (S.K.); hoerauf@uni-bonn.de (A.H.); 3 Institute for Medical Microbiology, Immunology and Parasitology, University Hospital Bonn, 53127 Bonn, Germany; 4 Department of Microbial Drugs, Helmholtz Centre for Infection Research, 38124 Braunschweig, Germany; miriam.grosse@helmholtz-hzi.de (M.G.); birthe.sandargo@helmholtz-hzi.de (B.S.); marc.stadler@helmholtz-hzi.de (M.S.); 5 German Center for Infection Research (DZIF), Partner Site Hannover-Braunschweig, 38124 Braunschweig, Germany; 6 Toxicological Consulting Services, 65795 Hattersheim am Main, Germany; ingo.stammberger@tcs-stammberger.com; 7 Institute of Pharmaceutical Biology, University of Bonn, 53115 Bonn, Germany

**Keywords:** toxicology, mesoporous silica, poorly soluble drugs, corallopyronin A, preclinical studies, vehicle

## Abstract

Toxicological studies are a part of the drug development process and the preclinical stages, for which suitable vehicles ensuring easy and safe administration are crucial. However, poor aqueous solubility of drugs complicates vehicle screening for oral administration since non-aqueous solvents are often not tolerable. In the case of the anti-infective corallopyronin A, currently undergoing preclinical investigation for filarial nematode and bacterial infections, commonly used vehicles such as polyethylene glycol 200, aqueous solutions combined with cosolvents or solubilizers, or aqueous suspension have failed due to insufficient tolerability, solubility, or the generation of a non-homogeneous suspension. To this end, the aim of the study was to establish an alternative approach which offers suitable tolerability, dissolution, and ease of handling. Thus, a corallopyronin A-mesoporous silica formulation was successfully processed and tested in a seven-day toxicology study focused on Beagle dogs, including a toxicokinetic investigation on day one. Sufficient tolerability was confirmed by the vehicle control group. The vehicle enabled high-dose levels resulting in a low-, middle-, and high-dose of 150, 450, and 750 mg/kg. Overall, it was possible to achieve high plasma concentrations and exposures, leading to a valuable outcome of the toxicology study and establishing mesoporous silica as a valuable contender for challenging drug candidates.

## 1. Introduction

During the preclinical drug investigations, an early toxicology assessment is required to ensure safe entry into human clinical studies [1]. This typically includes investigations in different preclinical species; however, these differ in physiologies and, thus, in requirements regarding the route of administration and the type of formulation [2]. Particularly for the development of poorly soluble drugs, providing an appropriate vehicle covering all requirements is an immense challenge for pharmaceutical scientists. In general, the oral administration is the most common route, which means that this must be considered during toxicology studies. Enabling sufficient exposure is crucial to identify adverse effects in conjunction with establishing a safety window for human administration. Solutions are the preferred formulations due to the easy handling (e.g., dose escalation) and the elimination of dissolution dependency [2]. However, in contrast to pharmacokinetic and pharmacodynamic studies, the dose levels required for a toxicology study exceed the therapeutically relevant dose many times over, with the aim of observing adverse effects. For drugs with low toxicity, the administered dose potentially increases to a maximum of 1000 mg/kg of bodyweight, which further limits the assortment of appropriate study vehicles [3]. Ideally, the vehicle should use a solubility-enhancing formulation strategy to provide improved exposure and, thus, high bioavailability. In addition, the successful evaluation of toxic effects is dependent on the tolerability of the respective vehicle, which is, in turn, dependent on species-specific factors such as route of administration tolerances, concentrations, volumes, dosing regimens, and study duration [4]. For example, the commonly used solvent polyethylene glycol (PEG) 400 is known to exhibit gastrointestinal adverse effects like watery feces or emesis following oral administration in dogs [5]. Enabling formulations like amorphous solid dispersions (ASD) are commonly used to improve bioavailability. However, high polymer quantities for several days can result in agglomeration and the formation of pharmacobezoars, leading to a fatal obstructive ileus [6]. The objective of this work was to identify an alternative vehicle for the toxicology studies in Beagle dogs for the low-toxicity anti-infective corallopyronin A (CorA), which is currently under late preclinical development for parasitic filarial nematode and bacterial infections [7]. According to the Guideline on Repeated Dose Toxicity, two different species of mammals should be used in preclinical drug development [3]. Previous toxicological studies were conducted in Wistar rats, representing the rodent species. For the toxicological investigation in a non-rodent species, Beagle dogs were selected. The combination of the poor aqueous solubility of CorA (91.13 µg/mL at pH 6.5) and the required high dose levels (150–750 mg/kg) excluded many common vehicles for toxicology studies [7]. Moreover, the waxy consistency of the fully amorphous CorA (glass transition temperature of 5 °C) did not allow for the suspending of the drug substance as a homogeneous suspension [7]. In recent years, mesoporous silica has been proven to be an easy-to-use powdered intermediate that increases the bioavailability of poorly soluble drugs. In contrast to ASDs, the risk of pharmacobezoar formation was assumed to be lower due to the mineral and inert character of mesoporous silica. Therefore, we aimed to introduce the mesoporous silica formulation principle as a drug carrier for toxicological studies of CorA and a potential option for future challenging drug candidates.

## 2. Materials and Methods

### 2.1. Materials

CorA (purity ≥ 90%) was produced by the Helmholtz Centre for Infection Research (Braunschweig, Germany) [7]. Mesoporous silica, Syloid^®^ XDP 3050, was kindly provided by Grace GmbH (Worms, Germany). Absolute ethanol (purity: 99.8%) was purchased from Carl Roth GmbH & Co. KG (Karlsruhe, Germany). NATROSOL^®^ 250 G Pharm was obtained from Caesar & Loretz GmbH (Bonn, Germany). For toxicokinetic studies plastic feeding tubes, sterile plastic syringes and needles for blood samplings were from B. Braun (Melsungen, Germany), blood sampling tubes Vacuette^®^ K2-EDTA were from Greiner Bio-One GmbH (Frickenhausen, Germany), and plastic reaction tubes were from Eppendorf SE (Hamburg, Germany). For the analytics of the plasma samples via HPLC, LC-MS grade acetonitrile, and water were purchased from Bernd Kraft GmbH (Duisburg, Germany), and LC-MS grade ammonium acetate from Merck KGaA (Darmstadt, Germany).

### 2.2. Methods

#### 2.2.1. Preparation of the Mesoporous Silica Formulation

The solid mesoporous silica formulation was prepared using the incipient wetness impregnation process [8]. CorA was dissolved in abs. ethanol with a concentration of 1 g/mL using an ice-cooled ultrasonic bath. Due to the small amount, the loading of the silica was conducted manually in a plastic mortar and pestle. The solution was added dropwise to the continuously mixed silica until a ratio of 2:1 (silica/CorA, (*w*/*w*)) was obtained. The resulting CorA-silica powder was dried at room temperature for 24 h in a vacuum oven (Binder GmbH, Tuttlingen, Germany) to remove the ethanol. The loading step was repeated to achieve the final ratio of 1:1 CorA-silica (*w*/*w*), corresponding to a drug load of 50%. Higher drying temperatures were impossible due to the thermal lability of CorA. As the maximum liquid to Syloid^®^ XPD/silica ratio of 1.65:1 reported by the manufacturer would have been below the required ratio of 2:1 liquid (CorA solution in ethanol 1 g/mL) to solid ratio (*w*/*w*), the loading was performed in a twostep process well below this value in order to enable the liquid load in a dry state [9]. However, the maximum liquid load was tested empirically via the testing of ascending ratios between liquid and silica. The final amount of added liquid was determined to the extent that a free-flowing powder was still present after the loading steps. The first loading was performed on a 0.97:1 liquid (CorA solution in ethanol 1 g/mL)/solid ratio (*w*/*w*). After an intermediate drying step (evaporation of ethanol), a second loading step was subsequently performed. After the final drying step, an overall loading efficiency of 89% ± 3% was obtained based on the extractable CorA amount from the loaded silica. The loading efficiency was defined as the extractable drug fraction out of the mesoporous silica. Key characteristics of the mesoporous silica and the CorA-mesoporous silica formulation are provided in the Supplementary Materials [10].

#### 2.2.2. Non-Sink Dissolution

Following the USP II dissolution, a miniaturized apparatus was established by Zecevic et al. [11]. The use of 20 mL of dissolution media represents a valuable tool in early drug product development, since small quantities of formulation can be tested, thus saving drug substance. The experiment conditions were set as follows: paddle speed: 75 RPM; temperature: 37 °C; medium: 0.05 M phosphate buffer (pH 6.8); dose: 5 and 10 mg, respectively. Previous dissolution studies of neat CorA and solubility-enhanced CorA-ASDs showed poor dissolution at pH 1 [12]. Similar results were expected for the CorA-mesoporous silica formulation. Since absorption usually takes place in the intestine, dissolution experiments were performed at pH 6.8. Online quantification was conducted using an 8453 UV/VIS spectrophotometer (Agilent, Waldbronn, Germany). Absorption was determined at a fixed wavelength of 307 nm, and the dissolution test duration was set to 3 h. The measuring interval was 3 min, and light scattering correction was integrated through the 3-point drop line method (450–550 nm). The experiments were carried out at least in triplicates.

#### 2.2.3. Toxicology Study Setup

Toxicology studies in dogs were performed at Aurigon Toxicology Research Center Ltd. (Dunakeszi, Hungary; study number: 799.313.6289) according to the Institutional Animal Care and Use Committee (IACUC) guidelines EU directive 2010/63/EU. The dogs were housed in humidity- and temperature-controlled rooms with an artificial light/dark cycle of 12 h. Food (SQC diet for dogs) and water (as for human consumption) were offered ad libitum. On treatment days, fasted animals (at least 16 h) were treated and food was distributed approx. 2 h after treatment. A total of 16 dogs (eight males and eight females) were treated in four dosing groups (vehicle control, low-dose: 150 mg/kg, middle-dose: 450 mg/kg, and high-dose: 750 mg/kg). Each treatment group was composed of two male and two female animals. The animals were treated with the CorA-silica formulation which was suspended immediately before administration in a 1%- NATROSOL^®^ solution. For each group the administration volume was 8 mL/kg, and the suspension was administered via an oral gavage. For the vehicle control group, the amount of silica correlated to the high-dose group of 750 mg/kg, i.e., a 6 kg dog received 4500 mg silica per administration. For the toxicokinetic evaluation, on the first day of the study, plasma samples were collected after 0.5 h, 1 h, 2 h, 4 h, 6 h, 8 h, and 24 h for the HPLC analysis and quantification of CorA. Necropsy was scheduled on day 8. The animals were anesthetized via intravenous injection (mixture of ketamine-xylazine-midazolam), and then exsanguinated and sacrificed.

#### 2.2.4. Toxicokinetic Analysis

CorA from plasma samples was quantified using HPLC with an Alliance e2695 separation module and a 2998 PDA detector (Waters, Eschborn, Germany). The collected blood samples were centrifuged for 10 min at 4 °C and 3220 g. The resulting plasma was mixed in a ratio of 1:3 with ice-cold acetonitrile and then vortexed for 10 s. Afterwards, the mixture was centrifuged for 25 min at 4 °C and 11,600× *g* and the supernatant was transferred into a HPLC vial. The HPLC conditions were set as follows: A sample volume of 5 µL was injected and quantified at a wavelength of 300 nm. A Waters XBridge^®^ Shield RP18 column (3.5 μm, 2.1 × 100 mm, 130 A) was used at 30 °C. Data were analyzed using the Empower 3 software FR2, and quantified using an external reference standard. A solvent gradient was used comprising mobile phase A (acetonitrile/water 5/95 with 5 mM ammonium acetate and 40 μL acetic acid per liter) and mobile phase B (acetonitrile/water 95/5 with 5 mM ammonium acetate and 40 μL acetic acid per liter) with a gradient of 70% A/30% B to 20% A/80% B, added stepwise within 30 min at a flow rate of 0.3 mL/min [7,13]. For toxicokinetic analysis, the plasma concentrations of each dog were analyzed with the PK-Plus^®^ software (Simulations-Plus, Lancaster, CA, USA) using a non-compartmental model.

#### 2.2.5. Solubility Determination in Biorelevant Medium

The solubility of CorA-silica was determined using the shake flask method for 24 h. The fasted state simulated intestinal fluid (FaSSIF-V2) and the fed state simulated intestinal fluid (FeSSIF-V2) were selected as well-known biorelevant media. An excess of CorA-silica was introduced into 10 mL of the respective biorelevant media and incubated for 24 h in a GFL 1083 shaking incubator (Gesellschaft für Labortechnik GmbH, Burgwedel, Germany) at 37 °C. For each medium, three flasks were prepared and analyzed. The withdrawn samples (1.0 mL) were centrifuged (5 min, 21,000× *g*, 37 °C) and the supernatant was diluted 10-fold with methanol and then quantified using HPLC (Section 2.2.4).

## 3. Results and Discussion

### 3.1. Non-Sink Dissolution of CorA-Silica

**Figure 1:**
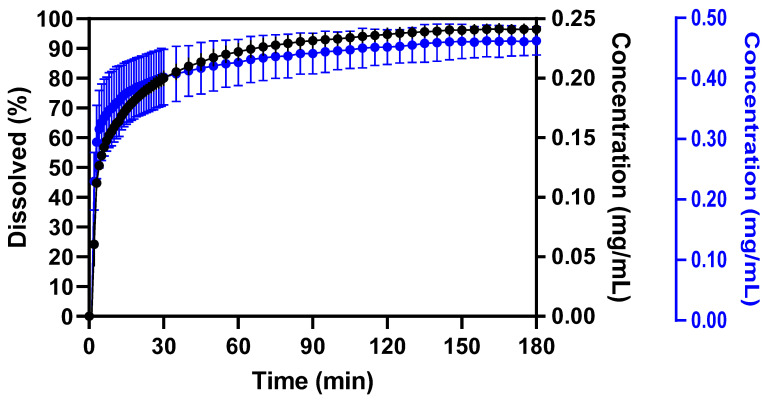
Mini-scale dissolution of the CorA-silica formulation (drug load 50%) (n = 3, mean ± SD). Experimental conditions were set as follows: Dose: 5 mg (●) and 10 mg (●); Volume: 20 mL 0.05 M phosphate buffer (pH 6.8); Paddle speed: 75 RPM; Temperature: 37 °C.

The dissolution performance of CorA-silica at pH 6.8 was studied using the mini-scale dissolution apparatus (Figure 1). Two target concentrations (0.25 and 0.5 mg/mL) were investigated to observe potential changes in the dissolution rate, depending on the drug concentration. A comparable performance was observed at both doses, with a fast initial drug release reaching 80% (0.25 mg/mL: ±3%; 0.5 mg/mL: ±9%) within 30 min. After 180 min, slight differences were detected as 97 ± 2% was observed for the lower dose, while the higher dose reached 93 ± 5%. This fast dissolution rate cannot be expected in the toxicological studies, as the doses of CorA exceeded those used for the dissolution experiments. However, the profiles indicated a faster drug release when compared to neat CorA [7]. Thus, this vehicle was expected to be suitable for toxicological investigations in dogs and tested in a seven day exploratory study.

### 3.2. Toxicokinetics

**Figure 2:**
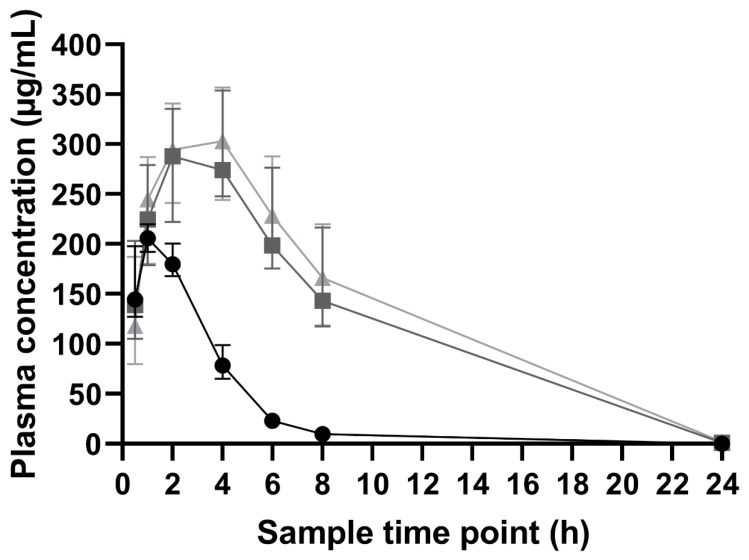
Toxicokinetic profiles of CorA-silica after oral administration in dogs (median ± IQR; *n* = 4 per group). The dose levels 150 mg/kg (●), 450 mg/kg (■), and 750 mg/kg (▲) were administered as a suspension in 1%-NATROSOL^®^.

The aim of a toxicology study is to identify potential adverse effects by administering doses higher than the therapeutic dose in order to provoke toxic effects in the time frame of the study, establishing a necessary safety window for the first in-human clinical trials. The maximum plasma concentration (C_max_) increased with ascending dose levels (Figure 2). No dose linearity was observed, leading to minimal differences between the middle and high-doses, which may be solubility and permeability limited. Thus, fast and high intestinal absorption did not increase in a dose-dependent manner. In contrast, pronounced increasing exposures (AUC_0-inf_) were observable with ascending dose levels, which suggested a permeation limitation for C_max_ (Table 1). Interestingly, between the low-dose and the middle-dose, the dose normalized exposure (AUC_0-inf per mg/kg dose_) increased slightly. The plasma concentration profile of the middle-dose led to a less steep elimination curve (Figure 2), indicating that CorA was still dissolving and absorbed after C_max_ was reached. Similar results were observed for the high-dose, which resulted in only a slight increase in the AUC_0-inf_. Consequently, the dose normalized exposure (AUC_0-inf per mg/kg dose_) for the high-dose decreased below the value of the low-dose (Table 1). It was assumed that at the high-dose level, intestinal solubility limited further dissolution as CorA was probably not absorbed fast enough. In general, the time where C_max_ was observed (T_max_) increased with an ascending dose level (2–4 h indicating a prolonged dissolution and absorption process).

By using the mesoporous silica formulation, it was possible to achieve increased solubility values for CorA tested in biorelevant media (FaSSIF: 0.835 ± 0.002 mg/mL; FeSSIF: 0.615 ± 0.031 mg/mL) [13]. Furthermore, in vitro dissolution indicated a fast dissolution rate. Although the maximum for the dissolvable drug seemed to be reached for the in vivo toxicokinetic trials, the undissolved part remained in the silica pores and served as a solubility-improved reservoir, which was able to replace absorbed drug molecules. For the low dose group, T_max_ was reached after 1 h. It was assumed that the reservoir function was rapidly exhausted, resulting in a rapid drop of the plasma concentration. In contrast, the T_max_ in the middle- (3 h) and high-dose (4 h) group were increased compared to the low-dose group (Figure 2), indicating a longer-lasting reservoir function accompanied with a sufficient concentration gradient.

Reservoir functions are already described in the field of ASD. Hirlak et al. demonstrated that a phase-separated colloidal drug phase is able to serve as a reservoir for already dissolved and absorbed drug molecules, thus improving the bioavailability of poorly soluble drugs [14]. Despite ASDs representing a promising approach for poorly soluble drugs to improve bioavailability, their use in toxicology studies remains limited. Gierke et al. identified the formation of pharmacobezoars in a nonclinical toxicological study in which rats were treated with a spray-dried hydroxypropyl methylcellulose acetate succinate (HPMC-AS)-based ASD [6]. Pharmacobezoars are agglomerates that form in the stomach and small intestine that can potentially lead to fatal obstructive ileus, depending on the dose and study duration. While HPMC-AS is a pH-dependent soluble polymer (soluble > pH 5), high amounts of polymer are also required to enable supersaturation, which in sum reinforces the formation of pharmacobezoars. Replacing the polymer may result in the impaired supersaturation or recrystallization of the drug within the ASD. Previous developed CorA-ASD formulations, comprised of povidone or copovidone, showed correspondingly promising solubility enhancements. However, the ASDs had a drug load of 20%. This would require a high amount of polymer, especially for the high dose level (600 mg/kg of polymer). The administration using capsules (size 0) would require an impractical number of capsules, approx. 50–100, depending on the individual body weight. Furthermore, providing a homogenous aqueous suspension is challenging due to the water-soluble ASD matrix and poor solubility of CorA. Therefore, an alternative was required to conduct toxicological studies of CorA in dogs. In contrast, mesoporous silica is assumed to be less prone to agglomeration due to its inert character. Moreover, the amount of silica required is lower due to the high loading capacities. In general, the physicochemical characteristics and physiological potency of each compound need to be considered, such as aqueous solubility, solvent solubility, the selection of appropriate polymers for an ASD and required ASD drug load, and the toxicological potential to guide the optimal vehicle selection. If only low doses are required for toxicological investigations, the use of ASDs could present a promising option. Even though mesoporous silica provided the best option for CorA, the use of alternative formulation principles may be beneficial for other compounds.

In comparison solutions, which, if possible, are the ideal approach for toxicology studies, solubility and dissolution are likely important in different ways for poorly soluble drugs. These drug candidates are usually dissolved in non-aqueous vehicles or solubilized with the help of excipients. After oral administration, the dissolved state can change rapidly and the drug may precipitate due to the limited solubility in the stomach or intestine [2]. The redissolving of the precipitate now determines the intestinal absorption. Therefore, a solution is not always able to guarantee full absorption during the toxicological study. The mesoporous silica approach, therefore, is not necessarily disadvantageous when compared to a solution. Another approach is the administration of an aqueous suspension. The formation of a homogeneous suspension is crucial to ensure a consistent dose application. It is often not possible to make a homogenous suspension of a sticky or agglutinated drug substance, as was the case for the sticky CorA. The mesoporous silica combined with a 1%-NATROSOL^®^ solution provided a homogenous and stable suspension, thus overcoming the handling issues caused by the consistency of CorA. Despite very high-dose levels, the interindividual variability was low, indicating a successful and consistent dose administration. Due to its aqueous insolubility and average particle size of 50 µm, it was assumed that the mesoporous silica is not absorbed in the intestine. This study clearly demonstrated mesoporous silica to be a valuable alternative in cases for which standard vehicles are not applicable. In addition, mesoporous silica was confirmed to not only improve handling, but also increase bioavailability, resulting in high exposures of CorA.

### 3.3. Clinical Observations

Although often suggested as an alternative to PEG 400, in a pilot study, the oral administration of PEG 200 to dogs also induced severe vomiting in four Beagle dogs. In contrast, the mesoporous silica vehicle was well tolerated by the dogs, as shown by the control group, in which no clinical symptoms were observed. Furthermore, the body weight and body weight gain of the control group animals were in a normal range during the in-life phase, and no decreased food consumption was observed. Overall, no abnormalities were observed regarding clinical observations, clinical chemistry, histopathology, organ weights, blood pressure, coagulation, or electrocardiograms. Thus, it was possible to clearly assign potential adverse effects of the groups treated with CorA to the drug under investigation. Consequently, mesoporous silica was suitable to provide a well-tolerated vehicle that enabled the high exposure of a poorly soluble drug for a toxicology study in dogs.

## Figures and Tables

**Table 1:** Toxicokinetic parameters of CorA-silica after oral administration in dogs (median ± IQR; *n* = 4 per group).

	AUC_0-inf_	AUC_0-inf_ per mg/kg Dose	C_max_	T_max_
	$\frac{µg*h}{mL}$	$\frac{µg*h*kg}{mL*mg}$	(µg/mL)	(h)
150 mg/kg	814.2	5.428	209.36	1
	(736.1–869.4)	(4.91–5.80)	(202.72–214.40)	(1–1.25)
450 mg/kg	2805.3	6.234	287.79	3
	(2735.4–3201.5)	(6.08–7.11)	(248.61–337.93)	(2–4)
750 mg/kg	3243.3	4.324	303.28	4
	(2856.2–3683.6)	(3.81–4.91)	(248.74–355.93)	(3.5–4)

## Data Availability

The data presented in this study are available in the research article.

## Supplementary Materials

The following supporting information can be downloaded at: https://www.mdpi.com/article/10.3390/pharmaceutics16030386/s1, Figure S1. Results of residual solvents determination (log scale) of the CorA-mesoporous silica formulation after manufacturing; n = 3 (mean ± SD); Table S1. Basic characteristics of Syloid^®^ XDP 3050 and CorA-mesoporous silica formulation; Figure S2. Representative Chromatogram Used for Quantifying Corallopyronin A Release from the Particles.
