# Supplementary material for: Mesoporous Silica as an Alternative Vehicle to Overcome Solubility Limitations
Source: Pharmaceutics. 2024 Mar 12;16(3):386. doi: 10.3390/pharmaceutics16030386 (PMC10975448; doi:10.3390/pharmaceutics16030386)
Supplement: Supplementary file 1 [file pharmaceutics-16-00386-s001.zip › pharmaceutics-2883271-supplementary.pdf]

Supplementary Materials

Mesoporous Silica as an Alternative Vehicle to Overcome Solubility Limitations

Tim Becker [1,2], Jan Heitkötter [1,2], Anna K. Krome [1,2,3], Andrea Schiefer [2,3], Kenneth Pfarr [2,3], Alexandra Ehrens [2,3], Miriam Grosse [4,5], Birthe Sandargo [4,5], Ingo Stammberger [6], Marc Stadler [4,5], Marc P. Hübner [2,3], Stefan Kehraus [2,7], Achim Hoerauf [2,3] and Karl G. Wagner [1,2,*]

[1] Department of Pharmaceutical Technology and Biopharmaceutics, University of Bonn, 53121 Bonn, Germany; tim.becker@uni-bonn.de (T.B.); j.heitkoetter@uni-bonn.de (J.H.); krome@uni-bonn.de (A.K.K.)
[2] German Center for Infection Research (DZIF), Partner Site Bonn-Cologne, 53127 Bonn, Germany; andrea.schiefer@uni-bonn.de (A.S.); kenneth.pfarr@ukbonn.de (K.P.); aehrens@uni-bonn.de (A.E.); huebner@uni-bonn.de (M.P.H.); skehraus@uni-bonn.de (S.K.); hoerauf@uni-bonn.de (A.H.)
[3] Institute for Medical Microbiology, Immunology and Parasitology, University Hospital Bonn, 53127 Bonn, Germany
[4] Department of Microbial Drugs, Helmholtz Centre for Infection Research, 38124 Braunschweig, Germany; miriam.grosse@helmholtz-hzi.de (M.G.); birthe.sandargo@helmholtz-hzi.de (B.S.); marc.stadler@helmholtz-hzi.de (M.S.)
[5] German Center for Infection Research (DZIF), Partner Site Hannover-Braunschweig, 38124 Braunschweig, Germany
[6] Toxicological Consulting Services, 65795 Hattersheim am Main, Germany; ingo.stammberger@tcs-stammberger.com
[7] Institute of Pharmaceutical Biology, University of Bonn, 53115 Bonn, Germany
* Correspondence: karl.wagner@uni-bonn.de; Tel.: +49-228-73-5271

S1. Methods

S1.1. Gas chromatography for Residual Solvent Assessment

Gas chromatography measurements were carried out to determine residual solvent after the preparation of the mesoporous silica formulations for toxicology studies. The amount of residual ethanol was determined using a Focus GC Gas Chromatograph, equipped with a flame ionization detector and TriPlus RHS Autosampler (ThermoFisher Scientific, Dreieich, Germany). Experiments were conducted under the following setup: Column: FS_CS_624 quartz capillary with 30 m length and an inner diameter of 0.32 mm; column surface: 6% poly-(cyanopropyl) phenylsiloxane and 94% poly-(dimethyl) siloxane. Samples of approx. 40 mg, accurately weighed, were transferred to 1.0 mL of phosphate buffer pH 6.8. After incubation at 80 °C for 10 min in the headspace oven, 1.0 mL gas volume was injected. The starting temperature of the column oven was set to 60 °C and gradually heated to 80 °C with a rate of 2 K/min following a 10 K/min up to 150 °C. Compressed air (2.0 mL/min) was used as carrier gas and nitrogen for a 1/5 split flow during injection. A calibration curve with five concentrations covering the range of the quantified samples were used for quantification.

S1.2. Loading Efficiency

To determine the loading efficiency, 10 mg of CorA-mesoporous-silica were added to 10 mL acetonitrile and continuous ultrasound was used for 1 h to induce drug release (ultrasound water bath Sonorex TK 52 H, Bandelin, Berlin, Germany). The withdrawn samples (1.0 mL) were centrifuged (5 min, 21,000 G, 37 °C) and the supernatant was diluted 10-fold with acetonitrile and quantified by HPLC. The HPLC conditions were set as described in section 2.2.4. The entire manufacturing and determination of the loading efficiency were performed on three batches.

S1.3. Particle Size

Determination of particle size distributions based on the laser diffraction measuring principle. A Horiba LA-960 laser diffractometer (Horiba, Kyoto, Japan) equipped with a red laser diode (650 nm; 5 mW) and a blue light emitting diode (405 nm; 3 mW) was used. Measurements of the mesoporous-silica formulation were carried out using the wet system technique, in which the sample is dispersed in a liquid. N-hexane was used as a dispersion medium that is not able to dissolve or swell the formulations or components thereof and has an appropriate low viscosity. The addition of 0.1% SPAN 80 reduced the surface tension and improved the wettability. Approx. 15 mL of this suspension was filled into a beaker and a small quantity of CorA-mesoporous silica was added. Continuous ultrasound was used for 30 s to break up residual agglomerates (ultrasound water bath Sonorex TK 52 H, Bandelin, Berlin, Germany). Afterwards the dispersion was introduced to the quartz cuvette of the laser diffractometer. Sustained mixing by a magnetic stirrer in the cuvette prevented re-aggregation and sedimentation. Three independent measurements were performed.

S2. Results

S2.1. Gas Chromatography for Residual Solvent Assessment

The residual ethanol as process solvent was determined after the second drying step to confirm compliance with the limit given by the authorities. Figure S1 illustrates that the final formulation had an ethanol concentration of 779 ± 147 ppm for CorA-mesoporous silica, far below the limit of 5000 ppm [1]. Consequently, the drying procedure was able to provide a product that fulfil the ICH guidelines.

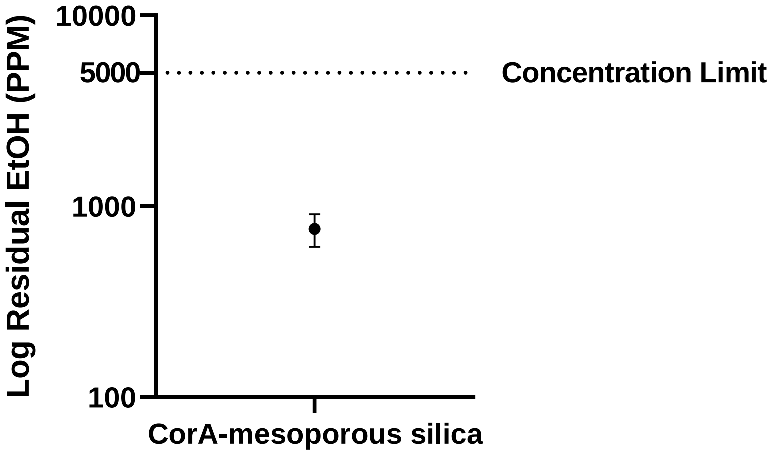

Figure S1. Results of residual solvents determination (log scale) of the CorA-mesoporous silica for-mulation after manufacturing; $n = 3$ (mean ± SD).

S2.2. Basic Characteristics of Syloid® XDP 3050 and CorA-mesoporous silica formulation.

Table S1. Basic characteristics of Syloid® XDP 3050 and CorA-mesoporous silica formulation.

Parameter	Syloid XDP 3050	CorA-mesoporous silica
Specific surface area (m^2/g)	320 [2]	
Pore size (nm)	22.9 [2]	
Pore volume (mL/g)	1.7 [2]	
Median particle size (μm)	59.42 ± 0.19 [2]	53.8 ± 0.5
Loading efficiency (%)		89 ± 3

S2.3. Representative Chromatogram Used for Quantifying Corallopyronin A Release from the Particles.

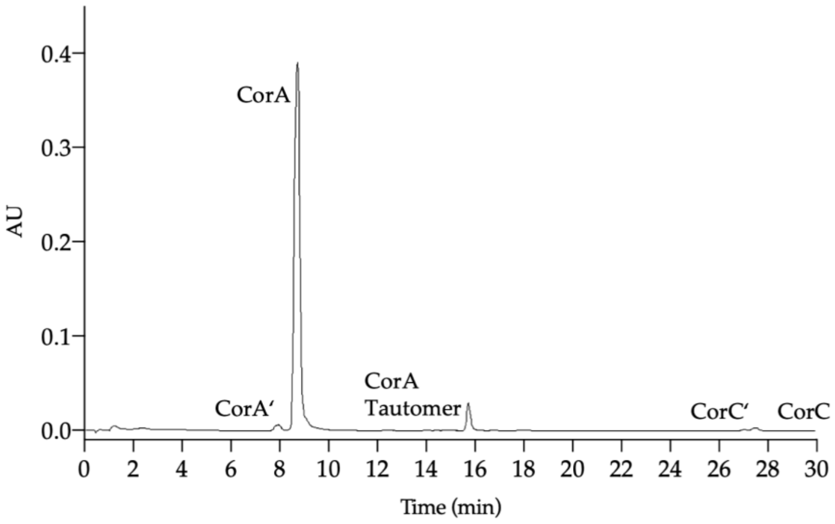

Figure S2. Representative Chromatogram Used for Quantifying Corallopyronin A Release From the Particles [3].

References

1. European Medicines Agency, ICH Guideline (Q3C (R8) on Impurities: Guideline or Residual Solvents. (2022) Online available: https://www.ema.europa.eu/en/ich-q3c-r8-residual-solvents-scientific-guideline (accessed on 24 February 2024).
2. Vraníková, B.; Niederquell, A.; Ditzinger, F.; Šklubalová, Z.; Kuentz, M. Mechanistic Aspects of Drug Loading in Liquisolid Systems with Hydrophilic Lipid-Based Mixtures. International Journal of Pharmaceutics 2020, 578, 119099, doi:10.1016/j.ijpharm.2020.119099.
3. Krome, A. K. Dissolution, Solubility and Stability Enhanced Formulation Strategies for the Novel Anti-infective Corallopyronin A, PhD thesis University of Bonn, 2021.
